# The Genotypic Structure of a Multi-Host Bumblebee Parasite Suggests a Role for Ecological Niche Overlap

**DOI:** 10.1371/journal.pone.0022054

**Published:** 2011-08-10

**Authors:** Rahel M. Salathé, Paul Schmid-Hempel

**Affiliations:** 1 Institute of Integrative Biology (IBZ), ETH Zürich, Zürich, Switzerland; 2 Department of Biology, Penn State University, University Park, Pennsylvania, United States of America; Biodiversity Insitute of Ontario - University of Guelph, Canada

## Abstract

The genotypic structure of parasite populations is an important determinant of ecological and evolutionary dynamics of host-parasite interactions with consequences for pest management and disease control. Genotypic structure is especially interesting where multiple hosts co-exist and share parasites. We here analyze the natural genotypic distribution of *Crithidia bombi*, a trypanosomatid parasite of bumblebees (*Bombus* spp.), in two ecologically different habitats over a time period of three years. Using an algorithm to reconstruct genotypes in cases of multiple infections, and combining these with directly identified genotypes from single infections, we find a striking diversity of infection for both data sets, with almost all multi-locus genotypes being unique, and are inferring that around half of the total infections are resulting from multiple strains. Our analyses further suggest a mixture of clonality and sexuality in natural populations of this parasite species. Finally, we ask whether parasite genotypes are associated with host species (the phylogenetic hypothesis) or whether ecological factors (niche overlap in flower choice) shape the distribution of parasite genotypes (the ecological hypothesis). Redundancy analysis demonstrates that in the region with relatively high parasite prevalence, both host species identity and niche overlap are equally important factors shaping the distribution of parasite strains, whereas in the region with lower parasite prevalence, niche overlap more strongly contributes to the distribution observed. Overall, our study underlines the importance of ecological factors in shaping the natural dynamics of host-parasite systems.

## Introduction

J.B.S. Haldane suggested in 1949 [1] that parasites might be responsible for the maintenance of genotypic variation in natural populations of hosts. In fact, the population genetic structure of micro-parasites is an important element for the ecological dynamics of a host-parasite system and of importance for practical problems, too. Examples include the conditions under which an epidemic can be controlled [2]–[4] the efficacy of interventions undertaken in agriculture animal husbandry and human public health [5],[6] or the general observation that host-parasite interactions are based on genotypic variation in both parasite infectivity and host susceptibility [7]. There is also a widespread consensus that genotype-genotype interactions have a major influence on host-parasite co-evolution [8],[9],[6].

So far, a number of ecological factors have been identified that affect parasite population genetic structure. For example, specific selection by the immune system can structure both parasite populations [10] and host heterogeneity [11], and host vaccination can potentially select for certain parasite types rather than others [12]. Also co-infections of hosts by parasites affect the parasite genetic structure in the host [13] and, similarly, structure depends on whether the parasites reproduce clonally or sexually, or show at least episodic genetic exchange among “strains”. This issue has been the focus of many debates over the past decades [14]–[16]. The range of these considerations is certainly relevant for the trypanosomatids, a representative of which is studied here. The group contains the agents of some major human diseases (e.g. sleeping sickness, Chagas disease, leishmaniasis) as well as important agricultural diseases, such as bovine Nagana fever and several plant diseases (caused by *Phytomonas*). Furthermore, in this group, genetic exchange has been demonstrated for *Trypanosoma brucei* [17],[18], *T. cruzi* [19], and *Leishmania major* [20]. Recently, we have been able to find experimental evidence for genetic exchange in *C. bombi* [21].

We here study the genotypic distribution of a contagious protozoan micro-parasite, the trypanosome *Crithidia bombi* [22] in field populations of its host, bumblebees *Bombus* spp., and in two distinct regions of Switzerland. Previous studies have repeatedly demonstrated the tight genotypic interactions between *C. bombi* and its model host *B. terrestris* [23],[24]. For instance, experimental transmission between colonies is affected by the genotype of the parasite as well as the genetic identities of both the targeted and the donor colony, and quantitative genetic studies have made it abundantly clear that host genotype is a major determinant of susceptibility to *C. bombi* [25]. As far as we know to date, *C. bombi* is restricted to hosts of the genus *Bombus*. Within *Bombu*s spp., *C. bombi* can be transmitted to other con-specifics inside the colony but also to any other potential con-generic host via visits of the same flowers [26]. Every locally present host species can potentially be infected, although the infection prevalence varies among species at a given site [27], [28].

Based on these data, we seek to shed light on the following questions: What is the basic genetic structure of the parasite populations, i.e. are populations locally structured and distinct from each other? How common are multiple infections in the field, and how genetically diverse are infections in general? Can we infer clonality or sexuality from analyzing the genotypic structure of these field infections? And finally, do parasite types associate with host species (the phylogenetic hypothesis); that is, is there segregation of parasite genotypes across different host species in the field, or are the parasite genotypes randomly distributed over all hosts that can harbor infections? Alternatively, can ecological factors like resource partitioning of host species, i.e. the dietary overlap at any given floral resource, affect the distribution of the parasite genotypes (the ecological hypothesis)? If the latter were true, we would expect that the different hosts would share parasites not only due to their genetic background, but also due to visiting the same flowers, i.e. the overlap of their ecological niches, and therefore the overlap in their infections - and *vice versa* if host species were the crucial factor. Note that flower preferences by foraging bees can be affected by a number of factors (e.g. tongue length, [29]–[31]. Yet here, we observe the realized flower preferences regardless of how they come about in order to assess possible pathways of transmission as a factor explaining the structure of the parasite populations. We collected data over a period of three years and in two regions representing typical bumblebee habitats of Central Europe. In particular, one region represents a habitat of relatively high infection prevalence of *C. bombi*, and the other represents a habitat with low parasite prevalence.

## Methods

### Sampling

Bumblebees were collected in two regions in Switzerland over a period of three years (2003, 2004, and 2005): (1) region “Basler Jura”, a hilly area (elevation 441–654 m) in Northwestern Switzerland, with the sites Röschenz (47°25′18″N, 7°27′22″E, 537 m), Soyhières (47°23′57″N, 7°22′13″E, 441 m), and Movelier (47°24′06″N, 7°19′15″E, 654 m); (2) region “Lower Engadin”, i.e. the Lower Engadin valley of the Southeastern Swiss Alps at elevations around the timberline (1'800–1'900 m), with the sites Buffalora (46°38′52″N, 10°16′00″E, 1'926 m), La Munt (46°38′20″N, 10°19′42″E, 2'205 m), Lavin (46°46′28″N, 10°6′36″E, 1'736 m), and Stabelchod (46°38′44″N, 10°14′26″E, 1'945 m), and one stretch just above the timberline, site La Schera (46°38′23″N to 46°38′42″N, 10°12′41″E to 10°14′48″E, at elevations of 2'300–2'584 m).

To ensure sampling an adequate range of infections throughout the season, we sampled several times during the season when possible, which for the Basler Jura could be further apart in total due to a longer season of flowering, i.e. late May, mid July and mid August. On the mountain meadows we sampled mid July and mid August, as the flower season at those altitudes is shorter by at least one month. For the sampling, arbitrary transects were defined in a site and all bees visiting a flower within a certain distance (2 m) to the left and right were collected. This approach was chosen to reduce sampling bias by randomly including all local landscape components instead of focusing on specific ones, e.g. a hedge or a hiking trail, to ensure the collection of *C. bombi* strain to be an adequate representation of the circulating parasites [32],[6]. After collection, all bees were freeze-killed and later screened for infections in the laboratory. To investigate the overlap of ecological niches for the different host species, the flower the collected bee had just been visiting was identified and registered; plant species was identified following the key of Hess et al. [33].

### *C. bombi* DNA extraction and microsatellite typing

We first dissected the bumblebees to check for parasites more generally under the stereo and light microscopes. Then, the bee guts (the site of infection of *C. bombi*) were extracted and individually placed into 1.5 ml Eppendorf tubes, together with 100 µl of Ringer solution (Merck); an equal volume of 10% Chelex (Bio-Rad) was added. The samples were heated to 95°C for 15 min, cooled on ice, vortexed and centrifuged for two minutes at 15'000 g. PCR was performed for five *C. bombi* specific microsatellite loci (labeled 4.G9, 4, 16, 2.F10, 1.B6) according to Schmid-Hempel and Reber Funk [34], with a standard PCR protocol: denaturation at 95°C for 5 min; 37 cycles of 1 min at 95°C, 30 sec at respective annealing temperature, 30 sec extension at 72°C; final extension at 72°C for 10 min. All products were visualized on Spreadex gels EL400 (Elchrom Scientific) and for increased accuracy loaded onto the 3130 ABI Prism Fragment Analyzer later on. Allele lengths were calibrated to the same standards across platforms.

Note that a new, co-infecting species of *Crithidia*, named *C. expoeki*, was recently described and is known to occur in the same regions [35]. However, the microsatellite primers used here do not amplify this new species. Therefore, we can safely say that our analysis only refers to populations of *C. bombi*.

### Algorithm to find *C. bombi* strains

An allele combination at a single locus defines a single-locus genotype. The combination of alleles over all typed loci is the multi-locus genotype, which is here heuristically considered a “strain”. A host can be infected with a number of genetically different strains at the same time. In this case it is not straightforward to separate these infections, i.e. to associate alleles with strains, and so to assess the number of concurrent infections. The reconstruction of constituent genotypes from multiple infections is a non-trivial problem, which has been considered not only in the field of biology, but also, for example, in applied forensic medicine [36],[37]. However, in situations like those analyzed here, where multiple host species and multiple infections of different strains of a parasitic species are involved, one cannot a priori rely on assumptions of Hardy-Weinberg frequencies or the knowledge of the genotypes of some suspects and reference persons, as it is done in forensic applications. Hence, for the time being, the problem of how to correctly reconstruct the constituent genotypes from field infections is not solved satisfactorily. To nevertheless use available data, and given that almost half of the hosts are multiply infected, we have used a conservative approach to reconstruct genotypes and calculate allele frequencies, and will report the results from single infections and all infections separately where appropriate. The algorithm used here is conservative as it gives priority to genotypes that are known from single infections, and since it assumes that infections with a maximum of two alleles at any one locus represent single infections. Schmid-Hempel and Reber Funk [34] employed this algorithm for the analysis of infections in spring queens (i.e. young queens emerging from hibernation and starting their colony); it is based on the diploid genotype of *C. bombi* and can be described as follows:

First, a minimum number of independent infections (n_min_) for all single loci in a given bee is calculated, as derived from the maximum number of alleles at any one single locus. Then, the maximum possible minimum number of strains infecting this bee is the largest value of n_min_ estimated from all loci. In a next step, the most probable circulating strains in the population are derived. These strains are identified from the singly infected bees in the sample (having no more than 2 alleles at any one locus). The respective multi-locus genotype is reconstructed from the unique combinations of alleles at all loci. These candidate single strains are then ranked according to their observed frequencies in the population, with the most abundant strain being the most likely candidate to be considered part of an observed multiple infection, and so forth for the less abundant strains, until the multiple infection is fully resolved. With this procedure, multiple infections can be partitioned into conservative combinations of strains that are most likely to make up the mixed infection in a given bee. This requires calculating all possible combinations of multi-locus types, and ranking them according to the ranking of their constituent single-locus types (for details, see [34]). We here used this algorithm to retrieve the strains from multiple infections.

It is not yet straightforward to partition a mixed infection into all its constituent, independent infection genotypes with certainty unless the infection is cloned and all clones are typed. Previous studies have pointed out the extreme difficulties of investigating complex mixtures of DNA probes [36],[37], and we emphasize, that the algorithm used here [34] provides only an approximate tool for the estimation of the constituent genotypes of mixed infections, and is purposely kept conservative to avoid overestimation. Hence, the true number of strains making up mixed infections is likely to be higher and similarly, the true prevalence of multiple infections is also likely higher, as is the number of unique genotypes, for the same reasons.

### Population genetic analysis and analysis of genetic distance

We first analyzed our data for the set of singly infected bees alone. In a second step, all infections were included, i.e. including the reconstructed strains retrieved from multiple infections as identified by the above algorithm. The overall population genetic structure was calculated using the software Genepop [38]. Pairwise F_ST_–values and their significance levels were calculated using Arlequin 3.1 [39]. Each directly identified or reconstructed multi-locus genotype was entered as one independent data point; populations with sample sizes smaller than 5 were not included in each of these analyses, and those with sample sizes lower than 10 are marked in italics. Locus 1.B6 was not included in the final analysis at all due to ambiguities in genotyping on the ABI Prism Fragment Analyzer (scattered ranges instead of clear peaks). We also tested for linkage disequilibrium according to the method of Black and Kraftsur [40] implemented in the software GENETIX [41].

In a further step, *C. bombi* populations for each site where bees had been sampled for consecutive years were pooled together into one data set for that given site. The resulting three parasite populations in the region Basler Jura (Röschenz, Movelier, Soyhières) and four parasite populations in the Lower Engadin (La Munt, Buffalora, Lavin, Stabelchod), respectively, were analyzed with respect to their phylo-geography using allele frequencies. Relationships were based on Cavalli-Sforza's and Edwards' chord distance D_C_ [42]. According to Takezaki and Nei [43], this distance is superior to other measures because it explicitly considers the stepwise nature of microsatellite mutation. Allele frequencies were bootstrapped 1'000 times, using the subprogram SEQBOOT within the program package PHYLIP 3.1. [44]; the consensus tree was assembled in NEIGBOR and CONSENSUS, and finally visualized in TREEVIEW [44].

At last, G∶N-ratios (number of different multi-locus genotypes, G, over sample size, N) were calculated as a measure of genetic diversity and clonality of *C. bombi* according to Ivey and Richards [45]. Calculations were done for all successfully typed multi-locus (4 loci) genotypes. Note that diversity at these four loci was high enough for the resolution to be appropriate for our analyses.

### Canonical redundancy analysis (RDA)

A redundancy analysis (RDA) was performed in the statistics package R version 2.10.1 (from the R Foundation for Statistical Computing) to look for correspondence between *C. bombi* alleles, i.e. parasite genotypes, and overlap in flower visits among different host species, and for the effect of host species itself. RDA [46] is a constrained principal component analysis and is related to canonical correspondence analysis (CCA; [47]). It can be used to compute a multifactorial and multivariate analysis of variance [48] and is the direct extension of multiple regression to the modeling of multivariate response data [49]. RDA examines how one set of variables (Y, for example, genetic variables such as the genotypes of the infection strains) may be explained by another set of variables (X, e.g. environmental variables such as overlap in flower visits and host species). In other words, the outcome of the analysis expresses how much of the variance in one set of variables can be explained by another set of variables. The input for the analysis is a set of transformed quantitative variables (Y) and any other variable (X), in our case a set of binary matrices for each set of variables, and the output are principal components of the residuals of X.

Results can be presented in a bi-plot, where the two dimensions represent the Y-variables, while vectors represent the X-variables. The longer a vector in the plot, the more variance is explained by the respective variable, X. Accordingly, variables that do not contribute much to explain the variance of the dependent data set are located in the center of the plot. The matrices we used are defined by (i) single alleles typed from the *C.bombi* infections, (ii) the independent single-locus genotypes (multi-locus genotypes could not be used, since each so defined genotype was almost unique in the sample), (iii) the bumblebee host species, and (iv) the flowers that these hosts had visited. Direct redundancy analysis was performed for each region as a whole, and for the single sites Movelier, Roeschenz and Soyhieres from the Basler Jura with the data set of 2004; these data sets produced enough residual components for complete analysis, which was not the case for all other data sets due to limited sample sizes for infected individuals per year and site. Since we have more than two sets of variables, a partial redundancy analysis had to be performed in addition to the direct redundancy analysis in order to control for the effect of a third set (Z) and to isolate the effect of data set X alone (for example to account for only the effect of flower visits while filtering out any effect of host identity). Finally, we used the ANOVA implemented for the RDA-analysis in R to test the output of the analysis for significance.

## Results

A total of n = 2'267 bees were sampled and scrutinized for all regions and years. A synopsis of data is given in Table 1 for each sampling site and year with the respective number of specimens sampled, the number of bumblebee species identified, the percentage of species infected at the respective site, relative total as well as multiple infection, the number of typed alleles for the four loci considered in the analysis, and the number of rare alleles found in the respective subpopulations (defined by their frequency ≤0.05.). The observations were very variable and distinct for each site, but in general infections in the lower, hilly region of the Basler Jura were clearly more common and genetically more diverse than in the alpine region of the Lower Engadin.

**Table 1:** Synopsis of sampling data and typed microsatellite alleles for each site and year.

Site	Bees^1^	Species^2^	Species infected^3^ (%)	Parasite Prevalence^4^ (%)	Proportion multiple^5^	Alleles (4.G9,4,16,2.F10)^6^	Rare alleles^7^
**Basler Jura:**							
Movelier 2003	48	6	33	6.25	0.33	3, 2, 1, 2	NA
Movelier2004	100	10	90	54	0.72	12, 7, 3, 7	11
Movelier 2005	58	12	50	27.59	0.31	7, 4, 3, 5	2
Roeschenz 2003	74	8	50	35.14	0.27	11, 5, 3, 6	7
Roeschenz 2004	166	7	86	55.42	0.58	11, 6, 3, 9	11
Roeschenz 2005	226	8	50	10.62	0.38	10, 4, 3, 6	3
Soyhieres 2003	80	8	38	6.25	0.20	7, 5, 3, 3	2
Soyhieres 2004	111	6	100	54.94	0.54	9, 6, 3, 6	6
Soyhieres 2005	150	8	63	12	0.44	8, 4, 3, 7	4
**Lower Engadin**:							
Buffalora 2003	92	9	33	7.61	0.29	5, 3, 3, 3	1
Buffalora 2004	191	11	55	6.28	0.50	2, 4, 3, 2	NA
Buffalora 2005	102	11	9	0.98	1	0, 1, 1, 3	NA
La Munt 2003	88	11	36	4.55	0.50	4, 4, 1, 4	1
La Munt 2004	193	13	62	12.44	0.71	10, 4, 3, 3	3
La Munt 2005	103	12	8	0.97	0	0, 1, 1, 1	NA
Lavin 2004	45	9	44	11.11	0	3, 4, 2, 3	NA
Lavin 2005	128	14	29	3.13	0	5, 3, 3, 2	1
Stabelchod 2004	194	13	31	5.67	0	2, 3, 2, 1	1
Stabelchod 2005	67	6	33	4.48	0.33	2, 3, 2, 2	NA
La Schera 2005	51	8	0	0	0	-	-

1 Number of bees collected (sample size).

2 Number of different host species in sample.

3 Percentage of species infected with *C. bombi*.

4 Percentage of infected individuals in sample.

5 Proportion multiple infections among all infections in sample.

6 Number of unique alleles typed for each locus; sequence of loci given in parentheses.

7 Total number of rare alleles found in sample.

### Multi-locus genotyping

Among all bees, a total of 310 individuals were infected by *C. bombi* in the Basler Jura (prevalence 30.60%, *n* = 1'013 bees samples in total), and a total of 82 infected bees were found in the samples from the Lower Engadin (prevalence 6.54%; *n* = 1'254 bees) for the entire period from 2003 to 2005. This data set includes all *Bombus* species found in the sampled communities. Typing the four chosen loci for *C. bombi* resulted in a total of 39 distinct alleles, 149 single-locus genotypes among all infections, and 213 distinct strains (i.e. distinct multi-locus genotypes). Of the total of 392 infections at least 173 (i.e. a fraction of 44%) were infections by multiple strains of *C. bombi* as revealed by the allelic pattern of the infection (i.e. the presence of more than two alleles for at least one locus). Note that the error rate in our typing of alleles due to PCR errors is small enough to be neglected - common Taq DNA polymerases are generally expected to have a base pair (bp) substitution error rate in the order of 8×10^−6^ point mutations per bp per PCR cycle [50], which cannot account for the diversity of alleles found here.

**Figure 1:**
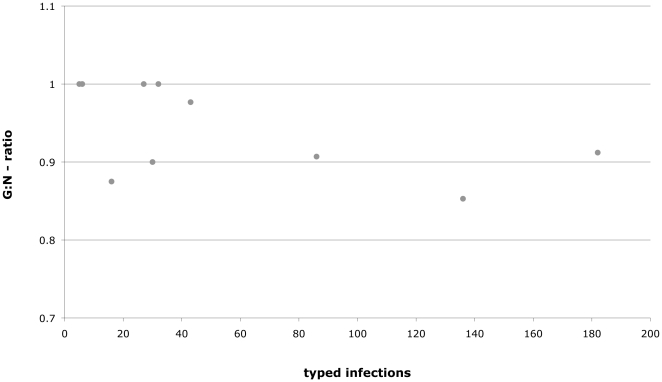
G∶N-ratios (number of genotypes over sample size) for all successfully typed multi-locus (4 loci) genotypes. Each dot is a different population. The typed infections on the x-axis (sample size *N*) represent the number of infections that were found and genotyped in the respective population.

Because of possible uncertainty involved in identifying the genotypes in multiple infections, we here present our analyses separately for single and for the pooled infections (single and reconstructed multiple infections). Our algorithm to reconstruct genotypes from multiple infections is conservative and will thus likely underestimate measures of Fst. Due to the limitations set by any marker-based system, only a finite number of different genotypes can be detected and additional genotypes will go unnoticed. We assessed the effect of such hidden diversity of genotypes by re-running the analyses including the first occurrence of the unique genotypes only. The results were virtually the same as when all cases were included (i.e. including the duplicate genotypes); this reflects the fact that almost every new infection contained a new genotype anyway (*c.f.* Fig. 1).

#### (a) Population genetics of single infections

*Population structure*. For all analyses we calculated F-values for the overall population of infections. Over all data sites and years, the F-values indicated a slightly structured overall population with a likely global excess of heterozygosity across sites and years (F_ST_ = 0.0285, p<0.001; F_IS_ = −0.0301, p = 0.064). The calculated number of migrants for the single infections are 16.77 (after correction for sample size; see [51], [52]), reflecting limited overlap between sites and regions. Considering the pairwise differentiation among the *C. bombi* populations at different sites for each year, we found widely varying F_ST_ -values, including significant separation of populations from one another, with pairwise F_ST_- values for all loci ranging from -0.146 to 0.1374 (see Table S1 in the Supporting Information). Together these results indicate that a substantial part of variation lies within the populations but that there is also variation between years of sampling at the same site. Testing for Hardy-Weinberg equilibrium (using the Markov chain method), of the 12 populations that were successfully analyzed, two significantly deviated from Hardy-Weinberg equilibrium (Table 2). Furthermore, there was no significant excess of heterozygotes over all populations (p = 0.146).

**Table 2:** *P*-values (S.E.) for the exact Hardy-Weinberg probability test (score U-test, for Markov chains with 1'000 iterations per batch, 100 batches) for each site and year, including separate sampling seasons.

Basler Jura Site	Single infections	All infections	Lower Engadin Site	Single infections	All infections
Movelier 2003	-	-	Buffalora 2003	*0.966*	*0.541*
Movelier 2004	0.887	**<0.001**	Buffalora 2004	*1.000*	**0.021**
Movelier 2005	0.393	0.237	early season	-	**<0.001**
Röschenz 2003	0.302	**<0.001**	late season	-	-
Röschenz 2004	0.320	**<0.001**	Buffalora 2005	-	-
early season	0.312	**0.011**	La Munt 2003	-	***0.014***
mid season	-	**<0.001**	La Munt 2004	*0.552*	**<0.001**
late season	0.451	**<0.001**	early season	-	0.977
Röschenz 2005	0.391	**0.009**	late season	-	**<0.001**
early season	-	**0.043**	La Munt 2005	-	-
mid season	0.786	0.102	Lavin 2004	*1.000*	*1.000*
late season	-	0.267	Lavin 2005	-	-
Soyhières 2003	-	*1.000*	Stabelchod 2004	**0.013**	**0.003**
Soyhières 2004	**0.001**	**<0.001**	Stabelchod 2005	-	-
Soyhières 2005	0.987	0.101			
early season	-	-			
late season	-	**0.008**			

Significant p-values are marked as bold. Data sets for which n<10 are marked italic; data sets with n≤5 excluded.

*Seasonal effects*. To assess for seasonal effects, we divided our datasets into seasonal subpopulations whenever possible, and performed the same Hardy-Weinberg tests as mentioned above. Due to restricted sample sizes, not all data could be used for this part, because we did not include instances with sample sizes of N<5. There was no substantial difference to the pooled data set (including all seasons) except that global heterozygote excess became slightly less supported (p = 0.175). Of the 15 successfully analyzed populations, only two (same as above) significantly deviated from Hardy-Weinberg equilibrium (at p<0.05). Seasonal subpopulations deviated from each other at various levels of significance.

*Linkage*. Among the total of 18 populations and 6 pairwise locus combinations, and using the complete data set for single infections only, we found 10 significant values for linkage (at p<0.05, Fisher's method; 6.5% of cases). Across all populations, linkage was significant for the locus pairs 4.G9 and 4 (p<0.001), and for loci 4 and 16 (p<0.01).

#### (b) Population genetics of all infections

Here, we make use of the reconstructed genotypes from multiple infections. As mentioned in the Methods section, the algorithm we used is conservative, i.e. it underestimates the number of different genotypes in the population. As a result, the measures of population structure (F_ST_) are likely to be underestimated, too. This needs to be appreciated in addition to an already inherent underestimation of differentiation by F-statistics found for data sets with high genotypic diversity [53].

*Population structure*. Over all data sites and years, and when including all infections, the F-values indicated a slightly structured population with global excess of heterozygosity across sites and years (F_ST_ = 0.0266, p<0.01; F_IS_ = −0.2050, p<0.01). The calculated number of migrants was 28.10, after correction for sample size as above. Considering the pairwise differentiation among populations at different sites for each year, we found widely varying F_ST_ -values, ranging from −0.746 to 0.3639 (see Table S2 in the Supporting Information). When considering all infections, 9 out of the 14 analyzed populations deviated from Hardy-Weinberg equilibrium (p<0.05) (Table 2), and there was strong heterozygote deficiency (p<0.001).

*Seasonal patterns*. We observed clear seasonal changes when considering all infections. For example, whereas the early subpopulation at Buffalora in 2004 significantly deviated from Hardy-Weinberg equilibrium (p<0.01), the late subpopulation was in perfect equilibrium (p close to one). For the subpopulations of La Munt in 2004 the situation was the opposite with a population starting out close to equilibrium in the early season (p = 0.98) and veering away from it in the later season (p<0.01). At Roeschenz in 2004 the subpopulation started out far from equilibrium (p = 0.01) and remained there throughout the season (p<0.001). In 2005 a different pattern could be observed at the very same site, with the subpopulations starting out far from equilibrium (p = 0.04), but slowly approaching it towards mid- (p = 0.10) and late season (p = 0.27). In the same year but at a different site (Soyhieres 2005), the pattern was again opposite, with the subpopulation starting out close to Hardy-Weinberg equilibrium early in the season (p = 0.91) and strongly deviating later in the year (p<0.01). Clearly, the genotypic infection patterns of these *C. bombi* field populations reveal a good deal of dynamic turnover varying among sites and from year to year. The study of multiple infections is thus especially helpful when elucidating seasonal patterns.

*Linkage*. For all infections, 81 possible tests produced 13 significant linkages (p<0.05, Fisher's method; 16.0% of cases). Across all populations, linkage was significant for the locus pairs 4.G9 and 4 (p<0.01), and for loci 4 and 16 (p<0.001), and 4.G9 and 2.F10 (p<0.001). Given that a total of 65 statistical tests are possible in this matrix, only the highly significant values might have a real basis, however.

#### (c) Genotypic correlations

Here, we include data from all infections because the bias towards lower diversity of genotypes in the set of re-constructed multiple infections does not change the conclusions, given that there is considerable diversity even among the single infections. Using all infections, we found that the number of genotypes relative to the number of samples (G∶N-ratios) varied from 0.853 to 1.0 for the 4-loci-genotypes (Figure 1). Most of the data points are above the 0.5-level, which according to Ivey and Richards (2001) is indicative of a bias towards sexual reproduction in the parasite population. The correlation between G∶N ratios and the host species diversity at a given site, H_s_, showed a negative but not significant trend (Spearman's rho = −0.451, p = 0.164). Figure 1 also shows that G∶N only slightly decreases as sample size increases, demonstrating that infections by *C. bombi* are extremely diverse in natural populations. The same holds true if only single infection were considered.

**Figure 2:**
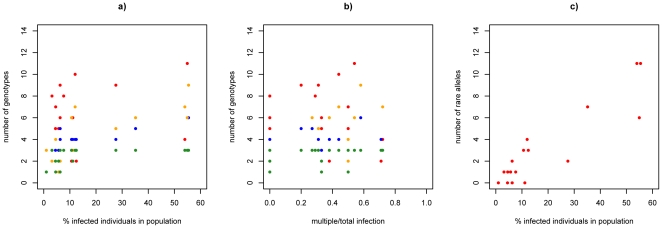
Correlation between a) the number of genotypes found in a population and the percentage of infected individuals in that respective population, b) the number of genotypes in a population and the fraction of multiple infections per total infections in that respective population, and c) the number of rare alleles found in a population and the percentage of infected individuals in that respective population. The respective loci are marked as follows: red = 4.G9, yellow = 2.F10, blue = 4, green = 16. (See text for statistics.)

Not surprisingly, there is a positive correlation between the percentage of infected individuals in a population with the number of *C. bombi* genotypes found in these respective populations for each locus (4.G9: R^2^ = 0.555, p<0.001, 4: R^2^ = 0.662, p<0.001, 2.F10: R^2^ = 0.635, p<0.001, 16: R^2^ = 0.235, p = 0.035) as well as for all loci pooled together (R^2^ = 0.344, p<0.001). Hence, the higher the infection load (prevalence of infections), the more genotypic diversity of the parasite population can be found (especially in Figure 2a). Different loci show this increase in different ways, with saturation quickly reached for the least polymorphic Locus 16. When correlating the genotypes found with the proportion of the multiple infections among all infection, there is a positive correlation between the number of genotypes and multiple infection for the more polymorphic loci, 4.G9 (R^2^ = 0.388, p<0.001), 4 (R^2^ = 0.384, p = 0.006), and 2.F10 (R^2^ = 0.352, p = 0.010), but there is no significant increase in genotypic diversity with increasing multiple infection for locus 16 (R^2^ = 0.115, p = 0.169) (Figure 2b). The regression over all loci together was positive (R^2^ = 0.203, p<0.001). The proportion of the total of rare alleles being found (with a frequency <0.05) also increases with increasing infection load in a respective population (R^2^ = 0.827, p<0.001) (Figure 2c).

#### (d) Genetic distance

**Figure 3:**
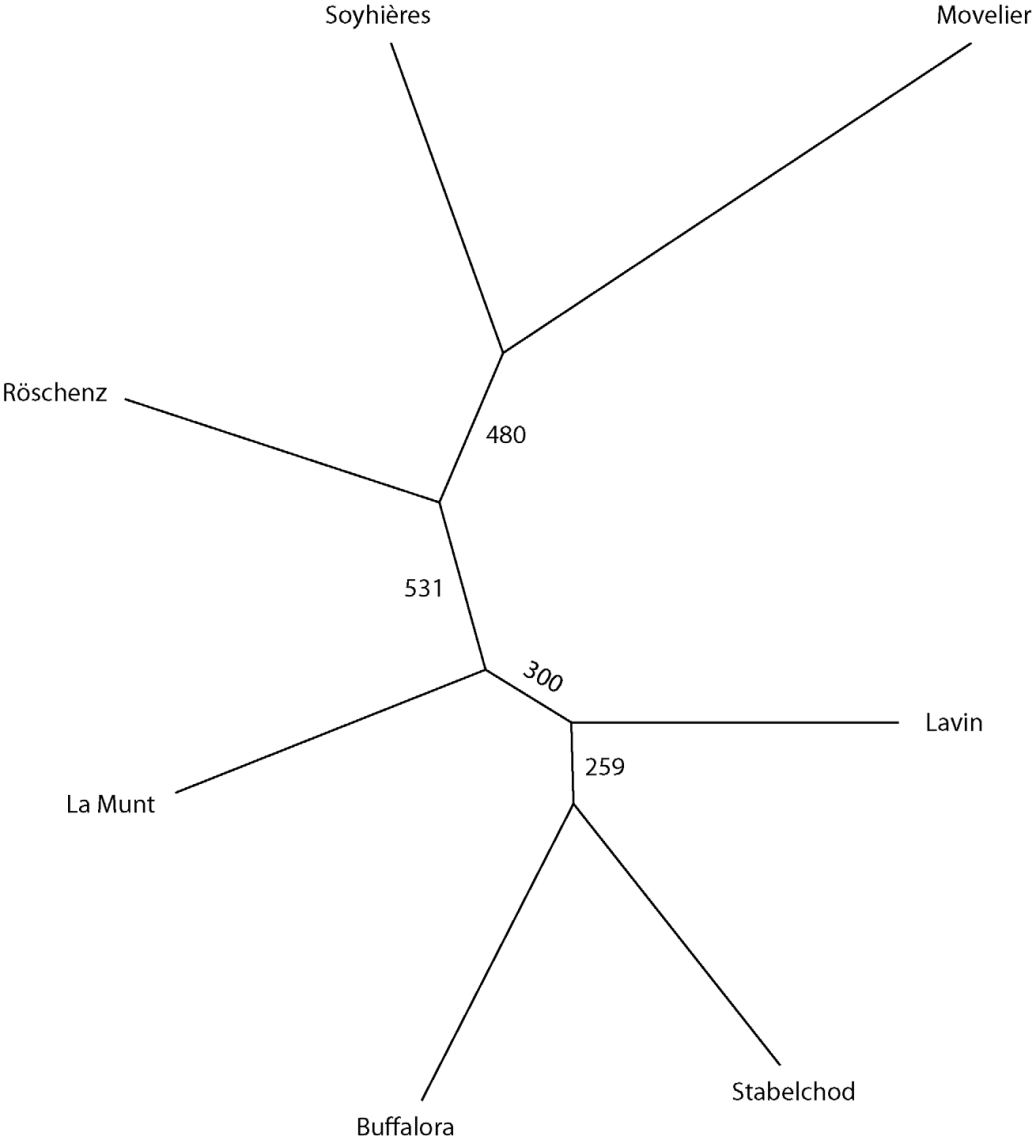
Genetic distance of *C. bombi* genotypes among the seven sampling sites. 1'000 bootstrap runs of an unrooted neighbor-joining tree were performed using Cavalli-Sforza's and Edwards' genetic distance. Small numbers indicate bootstrap values. The red line on the tree represents the geographic separation of the two regions Basler Jura and Lower Engadin. (Basler Jura: Röschenz, Movelier, Soyhières; Lower Engadin: La Munt, Buffalora, Stabelchod, Lavin.)

For the purpose of measuring genetic distances, we defined seven distinct geographic populations of *C. bombi* by pooling data sets from consecutive years for the same sites. This set was analyzed with respect to mutual genetic relationships between populations, i.e. their phylogeography. The resulting (un-rooted) tree corresponds to the geographic separation, with neighboring populations also being genetically close on the tree (Figure 3). The most supported branch in the tree (bootstrap value = 531/1'000) separates the two geographically distinct regions, the alpine (Lower Engadin) and the lower, hilly (Basler Jura) region. However, this support is still weak by the accepted standards. Among sites of the alpine region, *C. bombi* populations are genetically less divergent from each other than they are among sites of the Jura region. This may reflect the higher genetic diversification of *C. bombi* observed in the Jura region compared to the Alps.

### Niche overlap versus phylogeny

**Figure 4:**
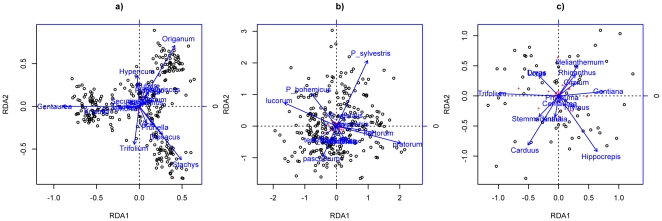
RDA (redundancy analysis) scatter plots for a) *C. bombi* flower visits constrained by host identity in the Basler Jura, controlled for the factor alleles (partial RDA, ANOVA F = 5.167, p = 0.005), b) *C. bombi* genotype segregation constrained by host identity in the Basler Jura, controlled for the factor flower visits (partial RDA, ANOVA F = 1.300, p = 0.005), and c) segregation of *C. bombi* genotypes constrained by flower visits in the Lower Engadin, controlled for the factor host identity (partial RDA, ANOVA F = 1.604, p = 0.005). The two axes illustrate the two principal components of the residuals of the multiple linear regression of X unto Y. The blue arrows designate the vectors for the constraining variable.

We tested whether host species identity (the phylogenetic hypothesis) or the overlap of flower visits (the ecological hypothesis), or both, are factors explaining the genotypic composition of the infecting parasite populations. We genetically characterized the parasite population by either the alleles at the various loci in separation, or as genotypes (combination of single infections and reconstructed multiple infections). Figure 4 depicts a subset of RDA plots with significant ANOVA outcome of the direct and partial redundancy analysis. The results varied among the regions and sites and sometimes significance levels depended on whether the allele matrix (i.e. independent alleles at loci) or the genotype matrix (i.e. single-locus genotypes, and reconstructed all genotypes) was used for the analysis (Table 3). For example, host species identity was the stronger predictor for both the parasite alleles and genotypes than the flower visits in the Basler Jura region. In the alpine Lower Engadin valley, host species identity was explaining most of the variation for the parasite alleles but not for the genotypes. On the other hand, when species identity is ignored, flower visit is a good predictor for parasite alleles and genotypes in the Lower Engadin and for the genotypes, but not for the alleles, in the Basler Jura. When separating sites and years, the patterns were not consistent within the respective region, which shows how dynamic the infections can change at any one specific location. With the exceptions of the Alpine sites Lavin and La Munt, where flower visit was a successful predictor, the factor species identity was a good predictor elsewhere. Note that as mentioned above, only the data sets with sufficient residual components could be used for complete analysis.

**Table 3:** Summary of redundancy analyses.

X_Y(_Z)	All data pooled (N = 377)	Lower Engadin (N = 70)	Buffalora (N = 20)	Lavin (N = 9)	La Munt (N = 27)	Stabelchod (N = 14)	Basler Jura (N = 307)	Movelier (N = 72)	Röschenz (N = 151)	Soyhieres (N = 84)
alleles_bees	**0.005**	**0.025**	0.090	**0.010**	0.210	**0.005**	**0.005**	**0.023**	**0.015**	0.580
alleles_bees(_flowers)	**0.010**	**0.010**	0.410	-	0.230	**0.015**	**0.040**	0.088	**0.045**	0.790
alleles_flowers	**0.020**	**0.005**	**0.005**	0.160	0.330	**0.024**	0.140	0.420	0.190	0.600
alleles_flowers(_bees)	0.105	**0.005**	**0.005**	-	0.350	**0.023**	0.530	0.650	0.320	0.620
genotype_bees	**0.005**	0.240	0.058	**0.010**	0.550	**0.015**	**0.005**	**0.005**	**0.025**	0.300
genotype_bees(_flowers)	**0.010**	0.115	0.085	-	0.190	**0.010**	**0.005**	**0.010**	0.240	0.860
genotype_flowers	**0.005**	**0.005**	**0.005**	0.130	**0.039**	**0.005**	**0.005**	0.056	**0.010**	**0.023**
genotype_flowers(bees)	**0.005**	**0.005**	**0.005**	-	0.210	**0.005**	**0.020**	0.520	0.054	0.270
bees_flowers	**0.005**	**0.005**	**0.010**	0.280	0.093	**0.030**	**0.005**	**0.005**	**0.005**	**0.005**
bees_flowers(_alleles)	**0.005**	**0.010**	0.270	-	-	-	**0.005**	**0.005**	**0.005**	**0.005**
bees_flowers(_genotype)	**0.005**	**0.005**	-	-	-	-	**0.005**	0.057	**0.005**	0.330
flowers_bees	**0.005**	**0.005**	**0.038**	**0.047**	0.380	**0.032**	**0.005**	**0.005**	**0.005**	**0.005**
flowers_bees(_alleles)	**0.005**	**0.020**	-	-	0.710	-	**0.005**	**0.005**	**0.005**	0.240
flowers_bees	**0.005**	**0.005**	-	-	0.490	-	**0.005**	0.230	**0.005**	0.800

Entries are the significance values for the particular combination of variables and sites. Y denotes the set of variables, which is explained by another set of variables X. A third set of Variables (Z) has been controlled for in a partial redundancy analysis in order to isolate the effect of X (with significant p-values in bold).

## Discussion

Our data yield the first quantitative estimate of the proportion of multiple infections of *Crithidia bombi* among all sampled infected bumblebee hosts. We found that from the total of 392 infected hosts whose infections were genotyped over the three years of sampling, 44% are representing multiple infections. This number is likely an underestimated considering that we had to reconstruct a portion of the genotypes, and potentially missed some actual diversity. Interestingly, the proportion of multiple infections presented here is similar to what Schmid-Hempel and Reber Funk [34] had reported for colonies of *B. terrestris* founded in the lab by spring queens caught in the field early in the season. A remarkable degree of diversity of different parasite genotypes had already been reported in that earlier study and, at least where more than two loci could be genotyped, no multi-locus genotype (“strain”) did overlap between colonies, indicating a very strong association of the distinguishable parasite genotypes with the different host colonies. Our data now demonstrate that in a field situation with several co-existing host species and the possibility of transmission among them, the diversity of parasite genotypes and the fraction of multiple infections is similar to the samples analyzed in the laboratory. This is remarkable because whereas the infection window for new infections had closed for the lab colonies at the moment of their isolation, the bees that forage in the field remain exposed to new infections continuously. Note that the fraction of multiple infections is higher in the denser parasite population in the Basler Jura than at the higher elevations, demonstrating the same effect.

The F_ST_-values reported here support the idea of segregation of the parasite genotypes across host individuals in the populations. Overall, each sampled *C. bombi* population at a given site in a given year appears to have its own dynamics, however. A recurrent observation is that the higher the prevalence of infection in a bumblebee community, the higher the number of alleles for the infecting population that is found (Figure 2). These high-infection populations also consist of more multiple infections, have a higher abundance of rare alleles, and deviate more from Hardy-Weinberg equilibrium. The variation in p-values resulting from the exact Hardy-Weinberg probability test (Table 2) also demonstrates the different dynamics in the different subpopulations, even more so when measuring at different time points, i.e. different stages in a season reflecting the buildup of the epidemic over the season.

The situation in *C. bombi* is perhaps not unusual, as varying population structures have also been reported, for example, for *Plasmodium falciparum* where epidemiological effects are suspected to affect the distribution of genotypes [54]–[56] and where the degree of clonality vs. sexual reproduction may vary considerably among vectors and populations [57]. Similarly, parasite population structure can vary with infection cycles (e.g. in *Trypanosoma cruzi* [58],[59]) and subtypes of a parasite can be associated with different phenologies and effects on the host (e.g. in *Leishmania infantum* [60], or *Trypanosoma brucei* [61]). Furthermore, strong selection by the variable host types can shape the population genetic structure of parasites in important ways [62] (e.g. in *L. donovani* [63]). Indeed, given the strong host-parasite interactions known from the *Bombus-Crithidia* system, a good (and as yet unknown) proportion of the parasite's population genetic structure should be determined by strong selection by the host, leading to a kind of the ‘iceberg effect’ [14] that makes only the varying small parts of the entire genotypic variation contained in single hosts visible to analyses.

As far as we understand the system, a bottleneck is imposed on the parasite population every autumn by the death of the worker and male host bees at the end of the season and the fact that only few colonies manage to produce young queens that carry the infection through hibernation [64]. Furthermore, the young queens vary considerably in their chance of successful hibernation. Therefore, we can expect that the genetic compositions of the spring parasite populations also vary considerably among species, colonies, and years. Also we find negative correlations between species abundance and the prevalence of *C. bombi* within and also between years of sampling and report more detailed ecological results elsewhere (Salathé & Schmid-Hempel, in prep). The building up of the infections over the season in turn might depend on a number of parameters, such as weather, host density, host species composition, environmental disturbance, floral abundance, and may specifically be affected by flower aggregation sizes determining foraging behavior of the hosts [65]. In the year 2004, for example, an exceptionally high prevalence of *C. bombi* infection was reported for all Jura sites (Movelier 54%, Röschenz 55%, Soyhieres 55%) and was followed by a “crash” in infection in the following season (Movelier 28%, Röschenz 11%, Soyhieres 12%), and a trend towards less deviation from Hardy-Weinberg, possibly indicating increased rates of events of genetic exchange among co-infecting parasite strains.

Unfortunately, it is almost impossible to track entire multi-locus genotypes for *C. bombi* across host species and years because the same multi-locus genotype is hardly ever found twice. Yet, it is feasible to analyze the individual alleles. For the three polymorphic microsatellite loci 4.G9, 4 and 2.F10, about half of the alleles found in this study are rare. Of the remaining alleles, one is usually the most common, although no systematic changes over the years can be confirmed, perhaps due to limited sample size. The increased occurrence of rare alleles with increasing infection prevalence (Figure 2) may indicate that more new combinations of alleles are generated by genetic exchange with increasing instances of co-infection. Linkage between alleles for each pair of loci varies from population to population, from year to year, and varies even within the locus pairs when only single infections are considered compared to all infections. This pattern may be a result of a mixture of clonal and sexual reproduction, similar to occasionally observed mixture of outcrossed and selfed offspring in hermaphrodites [66]. In fact, the diversity of the multi-locus genotypes found in the field (see Figure 1) could arise in a clonal population only with unusually high mutation rates, which we do not expect here. Furthermore, the G∶N values for *C. bombi* are definitely biased towards what is expected from sexual reproduction (Figure 1). We now know, in fact, that *C. bombi* strains regularly exchange genetic material, mostly following patterns of Mendelian segregation of parental alleles [21]. That study showed that in some cases alleles are lost or gained, leading to an entirely new genotype different from either parent.

The phylogenetic tree based on the typed microsatellites reflects the effect of geography on the distribution of *C. bombi* genotypes. Although the sites within each of the two regions were farther away from one another than a typical bumblebee would fly to forage, they roughly cluster together to form a geographic region. Considering that the bumblebee communities substantially differ in species composition between the two regions (personal observation), it is interesting to note that the distances in the tree are not correlating directly with the differences in host community structure. In La Munt, for example, the host species composition was similar to those in Stabelchod and Buffalora. Nevertheless, the distances in the tree between the sites La Munt and Buffalora are larger than between the two larger regions (see Figure 3). The proximity of Stabelchod and Buffalora on the parasite tree must therefore have a different cause than geography alone, and might be attributable to differences in the composition of the plant communities at these sites. Interestingly, the *C. bombi* populations of the alpine region are genetically less divergent from each other than they are within the sites of the Jura region. Whether this is due to a higher genetic diversification of *C. bombi* observed in the Jura region compared to the Alps, or whether it is a sample size effect would need to be investigated.

The importance of ecological factors for the population structure of the parasite is supported by the redundancy analysis (RDA) where, both, the species identity of the host as well as the overlap of foraging niches between hosts (shared flower visits) have an important effect on the distribution of *C. bombi* genotypes. The conclusions are somewhat different depending on whether the allele matrix or the genotype matrix is used for the analysis but the general message remains the same. Considering the Lower Engadin region, only the flower visits were a significant constraining factor to genotype distribution. Host identity does not seem to play such an important role in this region, despite the strong link of bumblebee species and flowers itself, and the fact that infected bees potentially carry the infection back to the colony. In all, it appears therefore that the population structure of infections might be driven by dietary overlap (the ecological hypothesis, which sets transmission patterns) that in turn is affected by species identity.

Given comparable cases, niche overlap between hosts has frequently been reported to enhance between-species transmission [67],[68]. *C. bombi* is a good representative for this scenario as it is readily transmitted horizontally via shared use of flowers [26]. In our study the ecological hypothesis is more strongly supported, presumably because transmission becomes more limiting. Studies including ecological parameters are therefore of great value for a better understanding of the dynamics of infections in the field, especially in a highly diversified system such as *Bombus* spp. and its parasite *C. bombi*.

## Supporting Information

Table S1 Pairwise F_ST_-values for single infections only for all sites and over all loci considered in the population genetics test (Arlequin 3.1, Excoffier et al. 2005). Significant p - values are marked as bold, for p≤0.05. Populations with sample sizes lower than 5 were not included in the test, values for data sets with n<10 are marked in italic. Population key: 1) Buffalora 2003, 2) Buffalora 2004, 3) La Munt 2004, 4) Lavin 2004, 5) Stabelchod 2004, 6) Movelier 2004, 7) Movelier 2005, 8) Roeschenz 2003, 9) Roeschenz 2004, 10) Roeschenz 2005, 11) Soyhieres 2004, 12) Soyhieres 2005.
(DOCX)

Table S2 Pairwise F_ST_-values for all infections for all sites and over all loci considered in the population genetics test (Arlequin 3.1, Excoffier et al. 2005). Significant p - values are marked as bold, for p≤0.05. Populations with sample sizes lower than 5 were not included in the test, values for data sets with n<10 are marked in italic. Population key: 1) Buffalora 2003, 2) Buffalora 2004, 3) La Munt 2003, 4) La Munt 2004, 5) Lavin 2004, 6) Stabelchod 2004, 7) Movelier 2004, 8) Movelier 2005, 9) Roeschenz 2003, 10) Roeschenz 2004, 11) Roeschenz 2005, 12) Soyhieres 2003, 13) Soyhieres 2004, 14) Soyhieres 2005.
(DOCX)
