# Supplementary material for: The Genotypic Structure of a Multi-Host Bumblebee Parasite Suggests a Role for Ecological Niche Overlap
Source: PLoS One. 2011 Aug 10;6(8):e22054. doi: 10.1371/journal.pone.0022054 (PMC3154203; doi:10.1371/journal.pone.0022054)
Supplement: Table S1 — Pairwise FST-values for single infections only for all sites and over all loci considered in the population genetics test (Arlequin 3.1, Excoffier et al. 2005). Significant p - values are marked as bold, for p≤0.05. Populations with sample sizes lower than 5 were not included in the test, values for data sets with n<10 are marked in italic. Population key: 1) Buffalora 2003, 2) Buffalora 2004, 3) La Munt 2004, 4) Lavin 2004, 5) Stabelchod 2004, 6) Movelier 2004, 7) Movelier 2005, 8) Roeschenz 2003, 9) Roeschenz 2004, 10) Roeschenz 2005, 11) Soyhieres 2004, 12) Soyhieres 2005. (DOCX) [file pone.0022054.s001.docx]

**Table S1**

1 2 3 4 5 6 7 8 9 10 11 12

1 0.00000

2 *-0.09681* 0.00000

3 *0.04165* *-0.05657* 0.00000

4 *-0.14672* ***0.08870*** *-0.00012* 0.00000

5 *0.01300* ***0.08641*** ***0.11806*** *0.03378* 0.00000

6 *0.03683* *-0.00283* *0.02239* -*0.07301* **0.07066** 0.00000

7 *0.03665* *0.04177* ***0.08221*** *-0.07004* **0.13737** 0.01469 0.00000

8 *0.02561* *0.02229* ***0.04912*** *-0.05362* **0.05980** -0.00445 **0.04836** 0.00000

9 *0.04577* *0.00498* ***0.03262*** *-0.05574* **0.07726** -0.01420 0.00370 **0.01852** 0.00000

10 *0.04167* *0.01765* *0.03228* *-0.02969* **0.10717** -0.00080 **0.04113** **0.03964** 0.01322 0.00000

11 *0.03380* *-0.00200* *0.03434* *-0.05038* ***0.07790*** *-0.01498 0.01529 0.00078 -0.00154 0.01053* 0.00000

12 *0.04796* ***0.05270*** *0.02693* *-0.06268* **0.10567** -0.02658 -0.00556 0.00119 -0.01623 0.00835 *-0.01237* 0.00000
